# Multimodal Diagnosis of Cardiac Amyloidosis: Integrating Imaging, Histochemistry, and Proteomics of Precise Typing

**DOI:** 10.3390/ijms27020820

**Published:** 2026-01-14

**Authors:** Jakub Kancerek, Łukasz Zniszczoł, Piotr Lewandowski, Romuald Wojnicz

**Affiliations:** Department of Histology and Cell Pathology, Faculty of Medical Sciences in Zabrze, Medical University of Silesia in Katowice, 41-808 Zabrze, Poland

**Keywords:** amyloidosis, histochemistry, immunohistochemistry, typing, cardiology

## Abstract

Amyloidosis is a group of disorders caused by extracellular deposition of insoluble fibrillar proteins, leading to progressive organ dysfunction. Cardiac amyloidosis is clinically significant, as myocardial infiltration results in restrictive cardiomyopathy, arrhythmias, and heart failure. The main subtypes are light-chain (AL) and transthyretin (ATTR) amyloidosis, while AA and isolated atrial amyloidosis (IAA) are less common. Accurate subtype identification is crucial for management and prognosis. Diagnosis requires a multimodal approach combining imaging and tissue-based techniques. Echocardiography is usually first-line, showing increased wall thickness, biatrial enlargement, and apical sparing. Cardiac magnetic resonance (CMR) provides superior tissue characterization through late gadolinium enhancement and elevated extracellular volume. Nuclear scintigraphy with 99mTc-labeled tracers enables non-invasive ATTR detection, while amyloid-specific PET tracers show potential for early diagnosis. Histochemical confirmation remains essential. Congo Red staining with apple-green birefringence under polarized light is the diagnostic gold standard, supported by Thioflavin T, PAS, and Alcian Blue stains. Immunohistochemistry and mass spectrometry aid amyloid typing, while electron microscopy provides ultrastructural confirmation. Integrating imaging, histochemical, immunohistochemical, and proteomic techniques enhances early recognition and precise classification, improving therapeutic strategies and patient outcomes.

## 1. Introduction

Amyloidosis is a heterogeneous group of diseases characterized by the extracellular deposition of insoluble fibrillar proteins, known as amyloid, in various tissues and organs. These fibrils are formed when normally soluble proteins misfold, adopt a beta-sheet structure, and aggregate into highly ordered, resistant fibers. Over time, amyloid deposits disrupt normal tissue architecture and organ function, leading to progressive organ damage. The disease can be systemic, affecting multiple organs, or localized, confined to a single tissue. The organs most commonly involved are the heart, kidneys, liver and peripheral and autonomic nervous system. Amyloidosis is classified according to the type of precursor protein involved. More than 30 different proteins have been identified that can form amyloid [1].

Cardiac amyloidosis is a condition characterized by the extracellular deposition of misfolded proteins, known as amyloid, within the heart tissue. These deposits can lead to structural and functional abnormalities, resulting in clinical manifestations such as heart failure, unexplained left ventricular hypertrophy, and, in some cases, severe aortic stenosis. The disease is most commonly caused by two major types of amyloid: immunoglobulin light chain (AL) amyloidosis and transthyretin (ATTR) amyloidosis, although other types, including AA and isolated atrial amyloidosis (IAA), can also affect the heart [2,3].

## 2. Amyloidosis

Light chain (AL) amyloidosis is a rare disorder, occurring in 5–10 people per million annually. It arises from plasma cell dyscrasias that produce misfolded immunoglobulin light chains. These light chains deposit mainly around cardiac cells, sometimes comprising more than 40% of the myocardial tissue. Deposition is often accompanied by inflammatory infiltrates, primarily T lymphocytes and macrophages, which exacerbate tissue damage and contribute to cardiac dysfunction [4,5].

Transthyretin (ATTR) amyloidosis results from the deposition of misfolded transthyretin, a tetrameric protein synthesized in the liver responsible for transporting thyroid hormone and retinol. It can be hereditary (hATTR), caused by mutations in the transthyretin gene, or wild-type (wtATTR), occurring without genetic mutations. Over 120 transthyretin mutations have been identified, with Val30Met being the most common, while Ile68Leu and Leu111Met are associated with a heart-dominant phenotype. Deposits of transthyretin typically appear as irregular nodules in the interstitium or along vascular structures. In wtATTR, the reasons for protein instability remain unclear, though age-related factors are considered contributors [1,6].

Serum amyloid A protein (AA) amyloidosis, also called secondary or reactive amyloidosis, is linked to chronic inflammatory conditions such as rheumatoid diseases. The amyloid precursor in AA amyloidosis is serum amyloid A (SAA), an acute-phase protein produced by hepatocytes in response to inflammatory cytokines. Cardiac involvement is rare, occurring in about 1% of cases, but when it does, it often manifests as right ventricular failure and indicates a poor prognosis. The kidneys are the most frequently affected organ, typically leading to proteinuria and hypertension [7].

Isolated atrial amyloidosis IAA results from the excessive production of atrial natriuretic peptide (ANP), a hormone involved in blood pressure regulation, natriuresis, and inhibition of the renin–angiotensin–aldosterone system. ANP and its precursor accumulate in the atria, particularly in conditions such as valvular heart disease, persistent atrial fibrillation, or after mitral valve replacement. While generally confined to the atria, these deposits can contribute to atrial structural remodeling and dysfunction [8,9,10,11].

## 3. Pathophysiology of Cardiac Amyloid Involvement

Cardiac involvement in amyloidosis results from the progressive extracellular deposition of misfolded protein fibrils within the myocardium, vasculature, and conduction system, leading to structural, metabolic, and electrical dysfunction [12]. In AL amyloidosis, myocardial injury arises from both physical infiltration of amyloid fibrils and the direct cardiotoxicity of circulating light chains, which induce oxidative stress, mitochondrial dysfunction, and activation of stress-related signaling pathways such as p38 MAPK and JNK, ultimately impairing contractility and promoting apoptosis; this dual mechanism explains the rapid clinical deterioration typical of AL disease [13]. In contrast, ATTR amyloidosis primarily causes myocardial stiffening through massive interstitial accumulation of transthyretin fibrils, which progressively thicken the ventricular walls, reduce compliance, and impair diastolic filling without the marked biochemical toxicity observed in AL [14,15]. Amyloid infiltration of intramural coronary arterioles produces microvascular dysfunction and reduced myocardial perfusion reserve, contributing to ischemia, myocyte degeneration, and worsening heart failure [16]. Involvement of the atrioventricular node and His–Purkinje system leads to conduction disturbances, including atrioventricular block, sinus node dysfunction, and atrial arrhythmias, particularly prominent in wild-type ATTR [17]. As deposition progresses, the extracellular matrix undergoes fibrotic remodeling with increased collagen deposition and expansion of the extracellular volume, further exacerbating stiffness and restrictive physiology [18]. Ultimately, the combined effects of mechanical infiltration, cellular toxicity, microvascular ischemia, and conduction system involvement culminate in severe diastolic dysfunction, low-output heart failure, and arrhythmic risk, defining the complex pathophysiology of cardiac amyloidosis [12].

## 4. Non-Invasive Diagnosis

Clinical diagnosis of cardiac amyloidosis relies on a multimodal imaging approach that integrates anatomical, functional, and molecular assessments. Transthoracic echocardiography is typically the first-line investigation due to its accessibility and ability to detect proper features, including increased left ventricular wall thickness, biatrial enlargement, restrictive diastolic filling, and pericardial effusion. Advanced techniques, such as longitudinal speckle-tracking strain imaging, can reveal the characteristic “apical sparing” pattern of global longitudinal strain, aiding differentiation from other hypertrophic phenotypes. Cardiac magnetic resonance imaging (CMR) offers superior tissue characterization, with diffuse subendocardial or transmural late gadolinium enhancement, prolonged native T1 times, and elevated extracellular volume serving as strong indicators of amyloid infiltration. CMR also supports disease staging and therapy monitoring. Cardiac scintigraphy with bone-seeking tracers labeled with 99mTc, such as 99mTc-DPD or 99mTc-PYP, demonstrates high myocardial uptake in most cases of transthyretin amyloidosis, enabling non-invasive diagnosis and subtype distinction without the need for biopsy in certain scenarios. Positron emission tomography (PET), although largely confined to research, utilizes amyloid-specific tracers like 11C-Pittsburgh Compound B, 18F-florbetapir, and 18F-florbetaben to visualize and quantify amyloid burden in vivo, offering promise for early detection and therapeutic response evaluation. Together, these modalities complement histological and proteomic methods, forming a comprehensive diagnostic framework that improves accuracy, facilitates amyloidosis subtype identification, and guides optimal clinical management [19,20].

## 5. Image-Guided Biopsy Strategy

While endomyocardial biopsy (EMB) remains the gold standard for definitive diagnosis, its sensitivity is intrinsically limited by the spatial heterogeneity of amyloid deposition. Standard transvenous biopsies are typically restricted to the right ventricular (RV) septum; however, amyloid infiltration can be patchy, particularly in early-stage disease or specific subtypes, leading to a risk of sampling error and false-negative results [21]. To mitigate this “blind” approach, pre-procedural imaging plays a critical role in predicting diagnostic yield. Cardiac Magnetic Resonance (CMR) provides a detailed non-invasive “roadmap” of amyloid distribution, specifically, late gadolinium enhancement (LGE) and T1 mapping can confirm whether the RV septum the intended biopsy site is actually involved [22]. If CMR reveals that amyloid deposition is confined to non-septal regions or is absent in the RV, the cardiologist can anticipate a low yield from a standard RV biopsy and may opt for alternative diagnostic strategies or direct the biopsy to more involved segments if technically feasible [23]. Thus, integrating CMR findings prevents futile procedures and enhances the precision of tissue sampling.

## 6. Biopsy-Based Diagnosis

Mass spectrometry (MS) is a powerful technique used to identify and characterize amyloid proteins, playing a crucial role in the diagnosis of amyloidosis. By measuring the mass-to-charge ratio of ionized molecules, MS provides precise information about the protein composition of amyloid deposits, confirming the presence of amyloid and helping to identify specific amyloid proteins, such as immunoglobulin light chains in AL amyloidosis or transthyretin in ATTR amyloidosis. Tissue samples are typically processed through laser capture microdissection or liquid chromatography before being analyzed by MS. While endomyocardial biopsy provides the most direct assessment, MS can also be successfully performed on specimens from surrogate sites, including abdominal subcutaneous fat, salivary glands, or the kidney and liver in systemic disease. This flexibility allows for precise typing even when cardiac tissue is not available, provided that amyloid deposits are present in the sampled surrogate tissue [24,25]. MS offers several advantages over traditional methods, such as its ability to detect multiple amyloid types in a single sample and its high sensitivity to small amounts of amyloid fibrils. However, its application in clinical diagnostics is limited due to the need for specialized equipment and technical expertise, making it more common in research settings or specialized centers.

Recent advancements in MS, such as tandem mass spectrometry (MS/MS) and MALDI-TOF (matrix-assisted laser desorption/ionization time-of-flight), have improved its accuracy and efficiency in identifying amyloid proteins. While MS provides definitive information about the protein composition of amyloid deposits, it is typically used alongside other techniques like Congo Red staining or immunohistochemistry to ensure comprehensive diagnostic evaluation, especially in complex cases such as cardiac amyloidosis [26,27]. While mass spectrometry (MS) represents the definitive method for amyloid typing at the molecular level, non-invasive imaging techniques play a pivotal role in the diagnosis, phenotyping, and longitudinal assessment of organ involvement, particularly in cardiac amyloidosis.

## 7. Histochemistry Techniques

**Figure 1:**
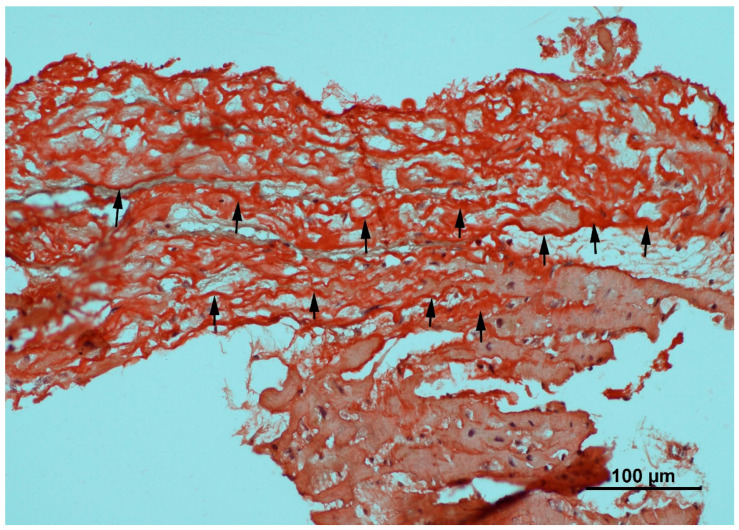
Congo red histochemistry stain of myocardial tissue under brightfield microscopy. The black arrows indicate the deposition of amyloid fibrils, which appear as characteristic brick-red or salmon-pink amorphous material infiltrating the interstitial space between cardiomyocytes. Note the distinction between the avidly stained amyloid deposits and the pale, unstained surrounding connective tissue and myocytes.

Congo red (CR) staining (Figure 1) is a histochemical technique used to detect amyloid deposits in tissue. The dye binds specifically to the β-sheet structure of amyloid fibrils, producing red staining under brightfield illumination and characteristic apple-green birefringence when viewed under polarized light between a crossed polarizer and analyzer. Tissue must be formalin-fixed, paraffin-embedded, sectioned (6–10 µm), deparaffinized, and rehydrated before staining. Hematoxylin may be added for contrast [28,29]. CR is highly specific for amyloid but cannot identify the protein subtype or quantify deposition. It may miss small deposits and occasionally produces background staining. For definitive diagnosis, CR is often combined with immunohistochemistry or mass spectrometry. Sensitivity can be improved using enhanced protocols, such as alkaline CR staining and digital analysis [30,31].

Crimson Red and Amyloid Red are histological dyes used as alternatives to Congo Red for detecting amyloid deposits. They bind specifically to amyloid fibrils, producing strong red or pink staining visible under brightfield microscopy. These dyes offer improved contrast over Congo Red, especially in cases with weak or ambiguous Congo Red staining, and are useful in complex amyloidosis. However, they may show non-specific binding and lack birefringence under polarized light, limiting their diagnostic specificity [32,33].

Alcian blue staining is a histological method used to detect acidic mucopolysaccharides and glycosaminoglycans in tissue, including components like mucins and sulphated proteoglycans. The dye binds to negatively charged groups, staining glycosaminoglycans at different pH levels: pH 1.0 for sulphated and pH 2.5 for both sulphated and nonsulphated forms. In amyloidosis, particularly AA type, Alcian blue helps visualize amyloid deposits rich in glycosaminoglycans. While not specific or primary for amyloid detection, it supports diagnosis when combined with other stains like Congo Red or immunohistochemistry [34,35,36].

Periodic acid–Schiff (PAS) staining is a histochemical method that detects polysaccharides, glycoproteins, and glycolipids by oxidizing vicinal diols to aldehydes, which react with Schiff reagent to yield a magenta color. In amyloidosis, PAS is useful for identifying deposits rich in carbohydrate components, particularly in AA amyloidosis where serum amyloid A associates with glycosaminoglycans. While not specific for amyloid, PAS can highlight Congo Red-negative or carbohydrate-rich deposits and help distinguish amyloid from other fibrillar materials. It is often used alongside Congo Red, immunohistochemistry, or mass spectrometry in the diagnostic workup [36,37].

## 8. Immunohistochemistry Typing

**Figure 2:**
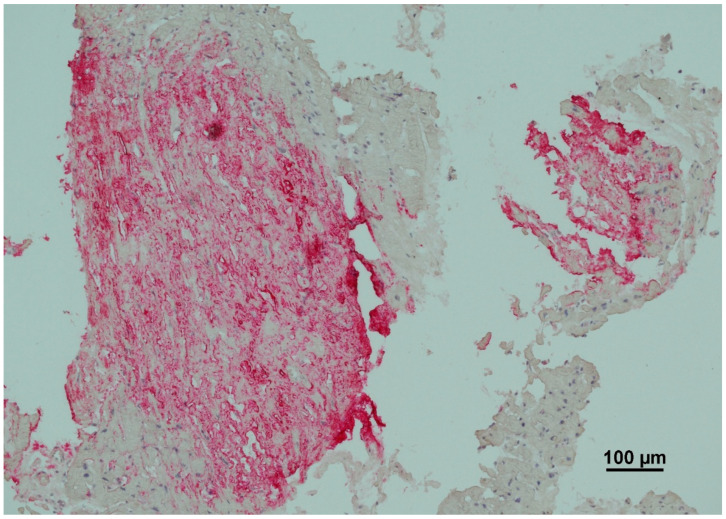
Immunohistochemistry (IHC) typing of AL amyloidosis. The red chromogen indicates positive antibody binding specifically to light-chain deposits within the amyloid nodule. The staining pattern is heterogeneous but specific, the intense red coloration is restricted to the amyloid aggregates, while the adjacent myocardial tissue remains unstained, confirming that the pattern represents specific antigen–antibody interaction within the infiltrates rather than a diffusion artifact.

Immunohistochemistry (IHC) is a key diagnostic tool in amyloidosis, allowing for the identification and classification of specific amyloid subtypes through antigen–antibody interactions. The technique uses monoclonal or polyclonal antibodies that bind to unique epitopes on amyloidogenic proteins, enabling targeted detection of amyloid deposits within tissue sections (Figure 2). The tissue is first fixed in formalin, embedded in paraffin, and sectioned into 4–6 µm slices. Following deparaffinization and rehydration, antigen retrieval, via heat or enzymatic digestion is performed to expose hidden epitopes [38,39]. Primary antibodies are applied to target specific amyloid types, such as light chains (κ, λ), transthyretin (ATTR), or serum amyloid A (AA). Secondary antibodies, conjugated to enzymes or fluorophores, bind to the primary antibodies and produce a signal-either chromogenic (color precipitate) or fluorescent (visible under a fluorescence microscope). IHC enables differentiation between amyloid types: κ/λ light chains for AL amyloidosis, transthyretin for ATTR, and serum amyloid A for AA. It also supports identification of rarer subtypes using antibodies against apolipoprotein A, gelsolin, or β2-microglobulin [40]. Despite its diagnostic value, IHC has limitations. These include antibody cross-reactivity, reduced sensitivity due to epitope masking from formalin fixation, and a limited antibody panel. It is also semi-quantitative and can be affected by inter-laboratory variability. However, recent improvements such as multiplex IHC, automated staining platforms, and digital pathology have enhanced the accuracy, consistency, and efficiency of amyloid typing using this method [41].

Thioflavin T staining is a fluorescence-based method for detecting amyloid fibrils. The benzothiazole dye binds to cross-β-sheet structures in amyloid, enhancing fluorescence emission at 482 nm when excited at 450 nm. After deparaffinization and rehydration of 4–6 µm tissue sections, samples are incubated with 0.1% Thioflavin T. Excess dye is rinsed off, and amyloid deposits appear as bright green fluorescence under a microscope (Table 1). It may show some binding to other β-sheet–containing structures like collagen [42,43].

Typing amyloid in cardiac muscle is a cornerstone of diagnosing cardiac amyloidosis, enabling the identification of the specific amyloid protein responsible for the disease. The typing differs, depending on variety of the disease. Methods that can come across are formed, depends on form of examination. In histological examination, various methods are employed to detect and type amyloid deposits in tissue samples [44].

## 9. Electron Microscopy

Electron microscopy (EM) is a high-resolution. technique It provides detailed ultrastructural images of amyloid fibrils, typically 7–12 nm in diameter, with a distinctive non-branching, filamentous morphology. Transmission electron microscopy (TEM) is the most widely used modality for visualizing amyloid fibrils in thin tissue sections. Tissue preparation for EM involves fixation with glutaraldehyde, post-fixation with osmium tetroxide, and embedding in resin. The sections are then stained with heavy metals such as uranyl acetate and lead citrate to enhance contrast. Under EM, amyloid fibrils appear as extracellular, organized structures often surrounded by cellular debris or matrix components [45]. Although EM is not routinely used in clinical diagnostics due to its technical complexity, cost, and time requirements, it is invaluable when other techniques, like Congo Red staining, provide ambiguous results. EM is crucial in research for understanding the architecture of amyloid fibrils and for identifying new amyloid types. Advanced methods like cryo-electron microscopy (Cryo-EM) and correlative light and electron microscopy (CLEM) further enhance its capabilities, enabling atomic-resolution imaging and combined imaging with other techniques to locate fibrils within tissues [46]. Despite its strengths in structural analysis, EM cannot directly determine the type of amyloid protein, requiring supplementary methods such as immunohistochemistry or mass spectrometry. Nonetheless, electron microscopy remains an essential diagnostic tool, particularly in complex cases like cardiac amyloidosis, where it provides invaluable structural insights when other methods are inconclusive [47].

## 10. Other Techniques

Laser Microdissection (LMD) is a precision technique used to isolate specific regions of tissue under microscopic visualization, enabling targeted molecular analysis. In amyloidosis, LMD is especially valuable for extracting amyloid-rich areas from heterogeneous tissue sections (e.g., heart, kidney, liver), allowing for high-purity samples suitable for mass spectrometry, PCR, or RNA sequencing. This targeted approach enhances sensitivity and reduces contamination, aiding in accurate amyloid protein typing especially important in mixed or sparse deposits. LMD supports research into amyloid genesis and disease mechanisms. While it requires specialized equipment and expertise, advancements in automation have improved its efficiency and broadened its clinical and research applications [48,49,50].

Formalin-fixed paraffin-embedded (FFPE) tissue analysis is a standard technique for diagnosing amyloidosis and other diseases, offering long-term preservation of tissue architecture for various analyses. In this process, tissue is fixed in formalin to maintain cellular integrity and embedded in paraffin wax, allowing for thin sectioning. FFPE samples are routinely used for histological staining, immunohistochemistry, and molecular methods such as PCR or mass spectrometry, which are essential for amyloid typing. Despite challenges in protein extraction due to cross-linking from formalin, advances in antigen retrieval and deparaffinization have significantly improved protein analysis. FFPE’s ability to utilize archived tissue from organs which makes it invaluable for both retrospective studies and routine diagnostics in amyloid diseases [50,51].

## 11. Current and Emerging Diagnostic Algorithms

Current diagnostic algorithms for cardiac amyloidosis increasingly favor non-biopsy strategies, particularly for transthyretin (ATTR) amyloidosis. The cornerstone of the non-invasive diagnostic pathway is bone-avid tracer scintigraphy (e.g., ^99mTc-PYP, DPD, or HMDP) combined with the exclusion of a monoclonal gammopathy via serum and urine immunofixation and free light chain assays; when imaging shows grade 2 or 3 myocardial tracer uptake and laboratory tests exclude clonal plasma cell disorders, a diagnosis of ATTR-CM can be made with high specificity without the need for endomyocardial biopsy [52,53]. Cardiac magnetic resonance (CMR) plays a complementary role, especially when scintigraphy results are ambiguous or in patients with rare TTR variants; advanced CMR techniques such as late gadolinium enhancement, native T1 mapping, and extracellular volume (ECV) quantification help characterize amyloid infiltration, assess disease burden, and monitor therapeutic response [54]. Emerging modalities include positron emission tomography (PET) using amyloid-specific tracers (e.g., ^11C-PIB, ^18F-florbetapir, ^18F-florbetaben), which allow for molecular quantification of amyloid load and may offer early detection before structural changes become apparent [54]. Recent refinements to diagnostic criteria also account for renal function when interpreting serum free light chains: for example, updated algorithms adjust κ/λ ratio cut-offs in patients with chronic kidney disease to reduce false positives, thereby improving the specificity of non-biopsy diagnosis [55]. In parallel, artificial intelligence and machine-learning approaches are being developed to integrate echocardiographic data, electronic health records, and laboratory parameters for earlier and more precise detection of cardiac amyloidosis. Taken together, these evolving diagnostic pathways reflect a shift toward less invasive, more sensitive, and personalized strategies, enabling earlier diagnosis and better monitoring in both AL and ATTR amyloidosis.

## 12. Future Directions

The trajectory of research and clinical management in cardiac amyloidosis is poised to be fundamentally influenced by advancements in diagnostic accuracy and therapeutic innovation. Emerging biomarkers, including circulating misfolded protein fragments, microRNAs, and comprehensive proteomic signatures, demonstrate substantial potential for the early detection of disease and for precise monitoring of progression or therapeutic efficacy [56]. Multimodal imaging platforms, integrating echocardiography, cardiac magnetic resonance, positron emission tomography, and artificial intelligence–driven analytic frameworks, are anticipated to enhance non-invasive diagnostic fidelity and enable quantitative assessment of myocardial amyloid burden in real time [57,58,59]. From a therapeutic perspective, the development of next-generation transthyretin stabilizers, gene-silencing agents, and gene-editing technologies holds considerable promise for transforming the management of ATTR amyloidosis [60,61,62], whereas targeted immunotherapeutic strategies aimed at neutralizing circulating amyloidogenic light chains or facilitating fibril clearance may substantially improve clinical outcomes in AL amyloidosis [62,63]. A deeper mechanistic understanding of amyloid fibrillogenesis, extracellular matrix remodeling, and microvascular compromise is essential to identify novel druggable targets [62,64]. The establishment of multicenter registries and prospective longitudinal studies will be critical to validate emerging diagnostic paradigms and therapeutic interventions, standardize clinical care, and facilitate the implementation of personalized treatment algorithms [59,62]. Collectively, the integration of molecular insights, advanced imaging modalities, and precision therapies has the potential to shift cardiac amyloidosis from a frequently underrecognized and progressive pathology toward a disease entity amenable to early diagnosis, accurate monitoring, and effective intervention [59,60,61,62].

**Figure 3:**
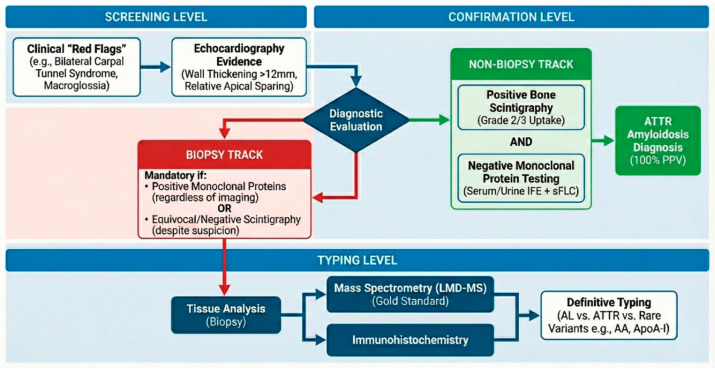
Hierarchical Multimodal Diagnostic Algorithm.

To establish a robust empirical framework for the clinical management of cardiac amyloidosis, it is necessary to move beyond isolated testing and adopt a hierarchical, multimodal diagnostic algorithm (Figure 3) that rigorously accounts for the sensitivity and specificity profiles of each technique. A systematic evaluation of diagnostic performance indicates that while echocardiography serves as the primary screening modality, its reliance on the characteristic “apical sparing” of longitudinal strain is limited by a pooled sensitivity of approximately 67% and specificity of 85%, largely due to morphological overlap with hypertensive heart disease and aortic stenosis [65,66]. However, diagnostic precision is substantially optimized when functional imaging is integrated with biomarkers; specifically, the incorporation of global longitudinal strain (GLS) with high-sensitivity troponin T into a multiparametric score increases the sensitivity for diagnosing AL amyloidosis to 94%, with a specificity of 97%, effectively filtering out non-amyloid hypertrophic phenotypes [67].

In the domain of advanced imaging, Cardiac Magnetic Resonance (CMR) provides superior tissue characterization through late gadolinium enhancement (LGE) and extracellular volume (ECV) mapping, achieving a sensitivity of 95% and specificity of 98% for detecting amyloid infiltration [54]. Nevertheless, meta-analyses underscore a critical limitation: CMR cannot reliably differentiate between AL and ATTR subtypes, with typing specificity falling below 60% due to the identical signal characteristics of the two fibril types [68]. Consequently, the diagnostic pathway for ATTR amyloidosis pivots to bone-seeking radiotracer scintigraphy, which utilizes calcium-mediated binding to TTR fibrils to achieve a sensitivity exceeding 99% [57,69]. Yet, a rational perspective necessitates the acknowledgment of false-positive risks—up to 22% of patients with AL amyloidosis demonstrate low-grade myocardial uptake, creating a dangerous potential for misdiagnosis if interpreted in isolation [70].

To mitigate this, the non-biopsy diagnostic criteria have been validated through large-scale multicenter cohorts, establishing that the presence of Grade 2 or 3 myocardial uptake yields a positive predictive value (PPV) of 100% for ATTR amyloidosis only when combined with the comprehensive serological exclusion of monoclonal proteins (via serum/urine immunofixation and serum free light chain assays) [53,71]. Clinicians must also remain vigilant regarding false positives arising from hydroxychloroquine toxicity, which induces vacuolar myopathy mimicking amyloid retention, and false negatives associated with specific mutations such as Phe64Leu or early-stage Val30Met variants, where scintigraphic sensitivity is markedly reduced [72,73,74]. In all discordant scenarios where imaging and serology do not align perfectly the algorithm mandates a reversion to the gold standard of tissue biopsy with laser microdissection and mass spectrometry (LMD-MS), which guarantees 100% specificity for amyloid typing and prevents catastrophic therapeutic errors [38].

## 13. Concluding Remarks

Cardiac amyloidosis constitutes a clinically heterogeneous and frequently underdiagnosed disorder associated with substantial morbidity and mortality. Contemporary insights into the pathophysiological mechanisms including extracellular amyloid deposition, cardiomyocyte toxicity, microvascular impairment, and conduction system disruption have delineated the biological basis for the diverse clinical phenotypes observed in AL and ATTR amyloidosis. The incorporation of advanced non-invasive imaging modalities, such as bone-seeking radiotracer scintigraphy, cardiac magnetic resonance, and emerging positron emission tomography techniques, in conjunction with circulating biomarkers, has refined diagnostic algorithms, permitting earlier and more accurate detection while potentially obviating the need for endomyocardial biopsy in appropriately selected cases. Prospective innovations, encompassing novel molecular biomarkers, artificial intelligence–assisted multimodal imaging, and targeted therapeutic strategies including transthyretin stabilizers, gene-silencing approaches, immunotherapies, and gene-editing interventions offer the potential for disease-modifying effects and improved clinical outcomes. Future investigations should prioritize the rigorous validation of these diagnostic and therapeutic modalities in multicenter, longitudinal cohorts, the development of patient-specific management algorithms, and the identification of additional molecular targets. Collectively, these advancements are poised to transform cardiac amyloidosis from a predominantly underrecognized and progressive entity into a condition amenable to early detection, precise monitoring, and effective therapeutic intervention, thereby improving both survival and quality of life.

## Figures and Tables

**Table 1:** Diagnostic Staining Patterns in Cardiac Amyloidosis.

	AL (Light-Chain)	ATTR (Transthyretin: wtATTR & hATTR)	AA (Serum Amyloid A)	IAA (Isolated Atrial Amyloidosis; ANP-Derived)
Typical cardiac deposit pattern	Predominantly pericellular or interstitial; deposits can occupy >40% of myocardium; often with T-cell or macrophage infiltrates that can obscure small deposits	Irregular interstitial nodules and vascular wall deposits; wtATTR often diffuse in older adults; hATTR varies with mutation (e.g., heart-dominant genotypes)	Cardiac involvement is rare (≈1%); when present, can involve right ventricle and portends poor prognosis; kidneys usually dominant organ	Atria (esp. in valvular disease or persistent AF; also after mitral valve surgery); atrial wall/appendage deposits
Thioflavin T	Bright green fluorescence when excited ~450 nm (emission ~482 nm) upon binding to cross-β fibrils; may show some signal with other β-sheet structures	Same fluorescence behavior; useful for highlighting diffuse interstitial fibrils	Positive where deposits exist; used as adjunct	Positive in atrial deposits
PAS	Magenta when carbohydrate-rich components present; helpful if CR is weak/negative in small foci	Variable; may be less conspicuous than CR/ThT unless matrix is carbohydrate-rich	Often conspicuous due to SAA association with carbohydrate/GAG components → stronger PAS signal than other types	Variable; depends on matrix composition, generally adjunctive
Congo Red (CR)	Brick-red on brightfield; apple-green birefringence under polarized light due to β-sheet binding; improved sensitivity with optimized (alkaline) CR protocols; background may occur in heavily inflamed tissue, so correlate	Same CR behavior as AL; typically robust birefringence in affected myocardium/vasculature	Standard CR positivity with birefringence when deposits present, but myocardial detection uncommon	Typical CR positivity with birefringence in atrial tissue
Alcian Blue	Blue staining of glycosaminoglycans; supportive rather than specific	Usually limited/auxiliary; presence reflects matrix GAGs rather than TTR itself	Can highlight acidic GAGs co-present in AA deposits (pH 1.0 vs. 2.5 helps subtyping of sulphated vs. non-sulphated GAGs)	Adjunctive
Alternative dyes (Crimson/Amyloid Red)	Strong red/pink on brightfield; no birefringence, so use as adjunct when CR is equivocal	Useful if CR suboptimal on section; interpret without polarization	Adjunct only	Adjunct if CR weak
IHC targets & typing notes	κ or λ light-chain antibodies support AL typing; beware epitope masking in FFPE and cross-reactivity; multiplex/automated IHC improves yield; MS remains gold standard when IHC is inconclusive	Anti-TTR antibody positive; IHC generally reliable but can be limited by fixation; consider mass spectrometry (LMD-MS) if antibody panel equivocal	Anti-SAA positive; use IHC panel given rarity of cardiac AA; confirm with MS if discordant	Anti-ANP (pro-ANP) immunolabeling supports IAA; important to sample atrial tissue—ventricular biopsies may be negative

## Data Availability

No new data were created or analyzed in this study. Data sharing is not applicable to this article.
